# Geroscience and Alzheimer’s Disease Drug Development

**DOI:** 10.14283/jpad.2023.103

**Published:** 2023

**Authors:** J. Cummings, A.M. Leisgang Osse, J. Kinney

**Affiliations:** Chambers-Grundy Center for Transformative Neuroscience, Department of Brain Health, School of Integrated Health Sciences, University of Nevada Las Vegas (UNLV), Las Vegas, Nevada, USA

**Keywords:** Geroscience, Alzheimer’s disease, senolytics, drug development, pipeline, aging

## Abstract

Age is the most important risk factor for Alzheimer’s disease (AD). The acceptable age range for participation in AD clinical trials is 50 to 90, and this 40-year span incorporates enormous age-related change. Clinical trial participants tend to be younger and healthier than the general population. They are also younger than the general population of AD patients. Drug development from a geroscience perspective would take greater account of effects of aging on clinical trial outcomes. The AD clinical trial pipeline has diversified beyond the canonical targets of amyloid beta protein and tau. Many of these interventions apply to age-related disorders. Anti-inflammatory agents and bioenergetic and metabolic therapies are among the well represented classes in the pipeline and are applicable to AD and non-AD age-related conditions. Drug development strategies can be adjusted to better inform outcomes of trials regarding aged individuals. Inclusion of older individuals in the multiple ascending dose trials of Phase 1, use of geriatric-related clinical outcomes and biomarkers in Phase 2, and extension of these Phase 2 learnings to Phase 3 will result in a more comprehensive understanding of AD therapies and their relationship to aging. Clinical trials can employ a more comprehensive geriatric assessment approach and biomarkers more relevant to aging at baseline and as exploratory outcomes. Greater attention to the role of aging and its influence in AD clinical trials can result in better understanding of the generalizability of clinical trial findings to the older AD population.

Alzheimer’s disease (AD) increases in frequency with aging and is accompanied by age-related brain and systemic biological changes in all but those individuals with young onset forms of the disease (1). From a pathobiological point of view, AD research has been most focused on amyloid, tau, and neurodegeneration (AT(N)) with additional emphasis on neuroinflammation (2, 3). Success in the development of anti-amyloid monoclonal antibodies has focused the field on amyloid as a target for disease modifying therapies (DMTs) (4, 5). Studies of the biology of aging have embraced a wider set of biological factors including stem cell exhaustion, epigenetic alterations, deregulated proteostasis, cellular senescence, mitochondrial dysfunction, inflammation, macromolecular damage, and adaptation to stress, many identified as the hallmarks of aging (6–9). The AD drug development pipeline is diversified and has agents targeting a variety of these age-associated biological processes (10). In this review, we examine the overlap between the biology of AD and biological changes of aging and explore how these overlapping processes may inform AD drug development. We highlight drugs in AD trials of special interest to aging, and we discuss “senolytics” that address cell senescence and their relationship to AD therapies.

## Geroscience and Alzheimer’s Disease Drug Development

Geroscience is an interdisciplinary field that studies the relationship between aging and age-related diseases and disabilities (11). Geroscience is based on the principle that the goal of biomedical research is to identify interventions that improve the quality of life in humans, and treating chronic diseases and conditions of the elderly represent an important pathway to achieving this goal (11). The geroscience approach notes that aging is the major risk factor for most chronic diseases including diabetes, hypertension, cardiovascular disease, cancer, and neurodegenerative diseases including AD. Medical science and drug development has traditionally approached one disease at a time, whereas the geroscience approach posits that aging contributes to essentially all chronic diseases and they commonly co-occur in the aging process. In clinical trials for AD, the occurrence of other age-related disorders in trial participants has been regarded as “comorbidity”, whereas it can be conceptualized as co-occurring disorders of aging and might respond to some AD therapies. Geroscience embraces the belief that aging is malleable with consequences for the lifespan, health span, and brain span when successful interventions for disorders of aging are found.

**Figure 1.**
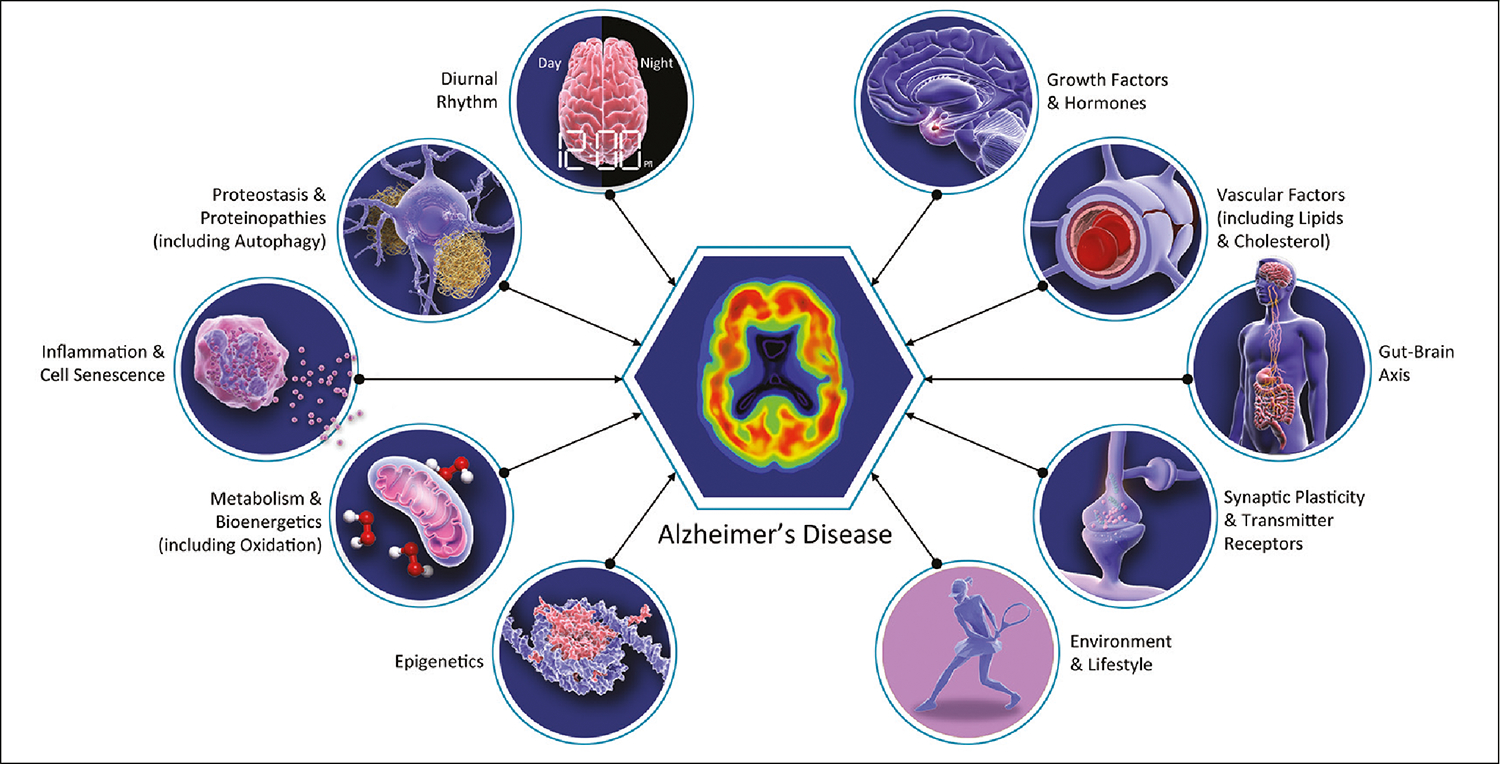
Biology of Alzheimer’s disease including many biological changes relevant to aging of multiple organ systems

The “geroscience hypothesis” states that interventions modifying aging biology can slow destructive aspects of aging resulting in the delay or prevention of the onset of multiple disorders (12). This hypothesis has profound implications for AD drug development; some AD therapies have mechanisms that might have effects across other age-related neurological disorders and other age-related systemic diseases. This suggests that a broader array of outcome measures is warranted in clinical trials of drugs whose primary target is AD but whose mechanism may apply to the biology of aging. Systemic benefits are particularly likely to occur when drugs being assessed in AD clinical trials relate to conditions known to be common in aging such as inflammation, oxidative stress, epigenetic changes, the microbiome, and mitochondrial dysfunction (Figure 1). Geroprotectors slow the rate of biological aging in animal models and may benefit aging in humans. Identification of geroprotectors that impact the brain could lead to novel therapeutics for AD, as aging is the greatest risk factor (13, 14). AD clinical trials frequently exclude patients with the more severe forms of age-related disorders, restricting the ability to understand the effects of AD drugs on aging conditions and limiting the generalizability of the clinical trial results to aging populations. Trial participants tend to be younger than real-world patients in the general population. This is due to challenges of recruiting and retaining older participants including fall risk, comorbidities and disability, transportation support, and attitudes of relatives and researchers towards older individual’s participation (15). A review of AD trial populations found that 78% of trial participants are under the age of 80 whereas 72% of AD patients are 80 or older (16). Adoption of a geroscience perspective in AD drug development may have implications for the agents chosen, approach to combination therapy, design of clinical trials, choice of biomarkers, and recruitment of trial participants.

## Drugs in Alzheimer Disease Clinical Trials with Mechanisms Related to Aging

Most agents in AD clinical trials have mechanisms that overlap with those of aging. In Table 1 we present the aging related agents that are in clinical trials for AD (Table 1) (10). We do not include drugs targeting amyloid beta protein, tau protein, proteostasis, or apolipoprotein E, whose mechanisms are more directly related to AD. Drugs relevant to aging in the AD pipeline include those aimed at cell death, abnormalities of the circadian rhythm, epigenetic regulators, growth factors and hormones, inflammation, metabolism and bioenergetics, neurogenesis, oxidative stress, synaptic plasticity, and vascular factors. These agents comprise 52% of the AD pipeline, demonstrating that approximately half of the drugs currently being tested in AD clinical trials might be expected to have effects beyond the central nervous system (CNS) and could impact multiple disorders of aging.

Areas within the AD/aging pipeline that are particularly well represented include inflammation (25 agents), synaptic plasticity (16 agents), and metabolism and bioenergetics (10 agents). The 25 unique agents targeting inflammation all target different aspects of the inflammatory process including microglial activation and cytokine release. Similarly, few of the drugs that targets synaptic plasticity have the same mechanism. Several of the drugs in the metabolism and biogenergetics category are derived from the repertoire of therapies developed for treatment of diabetes. Some of these agents, such as semaglutide, have both bioenergetic and anti-inflammatory effects.

Inflammation occurring as part of the aging process has been led to the concept of “inflammaging” as a key shared feature of chronic illnesses associated with aging (17, 18). Inflammaging refers to the chronic, sterile, low-grade inflammation which contributes to the pathogenesis of age-related diseases and arises from a variety of inflammatory stimuli that accompany aging, including pathogens, endogenous cell debris, misfolded proteins, and gut microbiota. Inflammaging contributes to accelerated aging, and successful intervention in this process may slow the destructive aging of multiple organ systems. Inclusion of clinical measures relevant to systemic inflammation as well as inflammation-related biomarkers (discussed below) in AD clinical trials assessing agents with anti-inflammatory effects may provide insight into their systemic and age-related effects.

## Therapeutic Categories of Special Interest for Alzheimer’s Disease and Aging

### Senolytics

Senolytics are a class of drugs developed to selectively eliminate senescent cells from aging organs (8). Senescence is a cell fate that is characterized by irreversible replicative arrest with sustained viability and resistance to apoptosis. Inducers of senescence include DNA damage, telomeric dysfunction, misfolded proteins and protein aggregation, decreased autophagy, reactive oxygen species, and inflammatory cytokines. Many senescent cells acquire a secretory phenotype that is proinflammatory, exacerbates inflammation occurring for other reasons, and accelerates biological aging. Senolytic drug development is challenging since there is no target receptor, enzyme, protein-protein interaction, or biochemical pathway. Nevertheless, drugs that appear to interfere with the sustained replicative arrest and allow senescent cells to be removed have been identified, shown to be of benefit in animal models relevant to senescence, and have entered clinical trials (8, 19).

There are 12 trials of senolytics currently registered on clinicaltrials.gov, a website where all clinical trials being conducted in the United States, and many clinical trials being conducted globally, are registered. All these trials are in Phase 1 or in an integrated Phase 1/Phase 2 clinical development program. Disease targets being explored in these trials include sepsis (1 trial); interstitial lung disease in individuals with immunodeficiency (1 trial); skeletal health (1 trial); non-alcoholic fatty liver disease (1 trial); chronic kidney disease (1 trial); mental disorders including schizophrenia, depression, and premature aging (1 trial); femoroacetabular impingement (1 trial); osteoarthritis (2 trials); and AD risk state, mild cognitive impairment (MCI), or AD dementia (3 trials). All three of the trials addressing AD are testing combinations of dasatinib and quercetin. Four of the non-AD trials are also testing this combination of agents. The remaining trials assess fisetin. Dasatinib is a tyrosine kinase inhibitor approved for the treatment of people with chronic myeloid leukemia and those with Philadelphia chromosome-positive acute lymphoblastic leukemia. Dasatinib and quercetin have been shown to ameliorate physical dysfunction and increase life span in aged mice (20). Quercetin and fisetin are flavonoid antioxidants with preclinical evidence of senolytic efficacy (21).

Outcomes in the AD risk trial include neurovascular coupling, executive function, gait speed, gait speed during a cognitive test, grip strength, and blood measures relevant to senescent cells. The trial assessing the dasatinib and quercetin in MCI is a Phase 1/2 trial in which safety and tolerability are the primary outcomes. In the Senolytic Therapy to Modulate the Progression of Alzheimer’s Disease (SToMP-AD) study, outcomes include adverse events, cellular senescence blood markers, Clinical Dementia Rating - Sum of Boxes (CDR-sb), Alzheimer’s Disease Assessment Scale - Cognitive Subscale (ADAS-cog), and tau positron emission tomography (PET). None of the other trials include assessments of cognitive function. In this set of trials of senolytic agents, assessments focus on a single disorder and do not include instruments that might capture more widespread effects on aging-related conditions or cognitive changes occurring in trial participants.

The trials of senolytics currently in progress do not reflect a geroscience perspective of common aging mechanisms across disease states that might be affected by interventions targeting common biological processes of aging. Inclusion of cognitive measures in trials of senolytics, and inclusion of measures of arthritis, skeletal function, and liver function in AD trials might enrich understanding of strategies for reducing accelerated aging. Only three drugs are currently being assessed in trials of senolytic agents (dasatinib, quercetin, fisetin). Assessing a wider array of senolytic drugs may allow insights into the impact on cellular senescence by agents with a range of mechanisms of action.

### Metformin

Substantial evidence supports the anti-aging effects of metformin. This agent is a biguanide originally introduced for the treatment of type 2 diabetes. Metformin has been shown in preclinical studies to attenuate the hallmarks of aging including improving nutrient signaling, enhancing intercellular communication, ameliorating proteostasis, protecting against genomic instability, regulating mitochondrial function, increasing stem cell rejuvenation capacity, regulating epigenetic alterations, minimizing telomere attrition, and attenuating cellular senescence (22). Observations in human populations (diabetic and non-diabetic) exposed to metformin provide evidence of increased longevity, decreased age-related diseases, and decreased all-cause mortality. Metformin has been observed to improve blood glucose control; reduce age-related musculoskeletal diseases including osteoporosis and intervertebral disc degeneration; ameliorate cardiovascular disease including heart failure and coronary artery disease; beneficially impact neurodegenerative disorders including AD, Huntington’s disease, and Parkinson’s disease; reduce obesity and fatty liver disease; and benefit polycystic ovary syndrome and chronic kidney disease (23).

The many observations supporting the beneficial impact of metformin on hallmarks of aging and age-related diseases led to the formulation of the targeting aging with metformin (TAME) study (22). A large number of potential biomarker outcomes for this study assessing many aspects of aging, frailty, and longevity have been proposed (24, 25). These biomarkers might be added to studies of AD clinical trials to add information about the impact of treatment interventions on age-related biomarkers (see discussion below).

Clinicaltrials.gov lists 28 interventional studies for metformin of which 11 are currently active. Targeted conditions in the ongoing metformin trials include diabetes, aging, muscle atrophy, frailty, impact on cesarean section scar, sarcopenic obesity, Parkinson’s disease, pulmonary hypertension, heart failure, cognitive decline in dementia, and MCI. The Phase 2 trial combining metformin with exercise includes the CDR, activities of daily living scales, blood pressure, body mass index measures, and weight circumference as outcomes. The combined Phase 2/3 study of metformin in MCI includes the following outcomes: Free and Cued Selective Reminding Test, Preclinical Alzheimer’s Cognitive Composite, cortical thickness measured by magnetic resonance imaging (MRI), white matter intensity volume measured on MRI, brain amyloid and brain tau assessed by PET, and plasma amyloid, tau, and neurofilament light. Two of the trials in which cognition is not the primary outcome have measures of cognition included among the repertoire of secondary measures being collected. Seven of the 11 active metformin trials have no cognitive assessment described on clinicaltrials.gov. There is an opportunity to increase understanding of the impact of metformin and similar agents on cognition by including cognitive and behavioral outcomes among the clinical trial outcomes in trials addressing targets unrelated to AD or cognition.

### Antioxidants

Oxidative stress is a common feature of aging and diseases of aging, and the production of reactive oxygen species may overwhelm endogenous antioxidant processes leading to oxidative injury. Oxidative damage has been linked to several age-related conditions including cardiovascular disease, chronic obstructive pulmonary disease, chronic kidney disease, cancer, and neurodegenerative disorders including AD (26). A variety of antioxidant therapies have been proposed to reduce oxidative stress associated with aging and oxidation-related accelerated aging. Candidate therapies include vitamins (β-carotene, vitamin C, vitamin E), coenzyme Q, selenium, polyphenols, flavonoids, and melatonin (26). As noted above the flavonoids are being explored for their senolytic potential.

### Vitamin E

High dose vitamin E was assessed in a double-blind placebo-controlled trial involving patients with moderate AD. The primary outcome was the occurrence of any of the following: death, institutionalization loss of ability to perform basic activities of daily living, or progression to severe dementia. Analyses that included adjustment for baseline severity scores showed a significant delay in time to primary outcome from 440 days to 670 days for patients receiving active vitamin E therapy (p = 0.001) (27). Reconsidering treatment with antioxidants using vitamin E or a related agent may be warranted in view of these findings. A contemporary antioxidant trial would use current diagnostic approaches, study conduct, clinical outcomes, and biomarker measures. Given the widespread occurrence of oxidative stress and oxidative injury in aging, measures of systemic effects of antioxidant therapy as secondary or exploratory outcomes are warranted.

### Taurine

Taurine is an essential amino acid that is derived partially from endogenous synthesis and partially from the diet. It exhibits antioxidant properties. Taurine’s major functions are within the mitochondria where it is required for synthesis of mitochondrially encoded proteins, reduces superoxide generation in the mitochondria, regulates intracellular calcium homeostasis, and inhibits mitochondrially mediated apoptosis (28). Taurine is being investigated for its possible therapeutic benefit in disorders of aging including cardiovascular disorders and mitochondrial disorders. Taurine dysmetabolism is implicated as a possible contributing factor to Parkinson’s disease and AD.

Valiltramiprosate (ALZ-801) is a prodrug of homotaurine and may be involved in taurine metabolism. Valiltramiprosate is being investigated for its therapeutic properties in AD patients with two copies of the apolipoprotein E4 gene. It is posited to reduce oligomerization and toxicity related to amyloid oligomers (29).

### Rapamycin

Rapamycin was the first agent observed to increase the lifespan of aged mice and greatly influenced studies of aging, since no drug had previously been shown to impact longevity in mammals. Rapamycin inhibits the growth of eukaryotic cells; the target of rapamycin (TOR) is a serine/threonine kinase. Rapamycin is approved by the US Food and Drug Administration (FDA) to prevent organ rejection in liver transplant patients, is used to prevent restenosis following coronary angioplasty, and is approved for the treatment of pancreatic cancer (30). Preclinical studies demonstrate increased longevity in a variety of strains of mice treated with rapamycin. Some studies have shown improved in cognition in both young and older mice treated with rapamycin (31). This agent has been associated with improved cardiac function, reduced rates of cancer, and beneficial effects on AD, Down syndrome, Huntington’s disease, and Parkinson’s disease (30).

Two studies of rapamycin are listed on clinicaltrials. gov. One is a study of rapamycin penetration of the blood brain barrier over an 8-week period in 10 patients. The other is a trial of rapamycin in MCI or mild to moderate AD dementia confirmed with amyloid imaging. This is a 12-month trial involving 40 patients. Primary outcomes are drug-placebo differences in adverse events and serum electrolytes. Secondary outcome measures include cerebrospinal fluid (CSF) levels of rapamycin and drug-placebo differences in CSF amyloid, glucose metabolism measured on fluorodeoxyglucose PET, and brain volume measured by MRI. Secondary cognitive outcomes include drug-placebo differences on the Preclinical Alzheimer’s Cognitive Composite, CDR-sb, Functional Assessment Scale, Geriatric Depression Scale, gait speed, and grip strength. This study may provide information regarding clinical and biomarker outcomes following treatment with rapamycin.

### Epigenetic Therapies

Epigenetic alterations include DNA methylation through reduced levels of the core histones and altered patterns of histone post translational modification; such alterations commonly accompany aging (32). These changes are observed in aging cells and in senescent cells. Epigenetic changes lead to aberrant gene expression and genomic instability. Epigenetic alterations are influenced by diet and environmental factors and account for some aspects of the heterogeneity of aging. Interventions aimed at epigenetic alterations include caloric restriction and histone deacetylase inhibitors.

### Sirtuins

The sirtuins (SIRTs) constitute a class of histone deacetylase or adenosine diphosphate-ribosyltransferase inhibitors that is nicotinamide adenine dinucleotide (NAD+)-dependent. Sirutins are implicated in the longevity effects of caloric restriction. Reduction of caloric intake is believed to slow aging by increasing sirtuins through stimulation of adenosine monophosphate-activated protein kinase (AMPK), thus raising the level of intracellular NAD+ by enhancing NAD+ biosynthesis (33). Seven SIRT family members have been identified in mammals. SIRT1 has been studied for its role in vascular aging. SIRT3, SIRT4, and SIRT5 are active in mitochondria (34). SIRTs regulate cell energetics and metabolism through mitochondrial effects and influence brain insulin signaling. Sirtuins are influential in amyloid and tau metabolism and in neuroprotection (35). Sirutins regulate neuroinflammation and inflammation-related neurodegeneration (36).

Effects of resveratrol have been shown to be SIRT-dependent (37). A double-blind placebo-controlled trial of resveratrol in mild to moderate AD showed no clinical benefit on cognitive and global measures. There was a nominally significant improvement of activities of daily living in favor of active therapy. Biomarker findings (MRI brain volume and plasma and CSF amyloid measures) did not provide definitive insight into biological effects of therapy (38).

## Bromodomain and Extraterminal (BET) Inhibitors

Another approach to histone deacetylase inhibition is through the bromodomain and extraterminal (BET) proteins that bind to acetylated histones leading to an amplified inflammatory response. BET inhibitors are small molecule epigenetic modifiers that bind to the bromodomains of BET proteins preventing their interaction with acetylated histones. BET proteins bind to acetylated histones and may lead to aberrant gene expression with amplification of inflammation and other epigenetic irregularities. BET inhibitors may be anti-inflammatory agents with therapeutic potential for treatment of many disorders including neurodegenerative diseases and AD.

Apabetalone is an oral small molecule inhibitor of BET proteins. Apabetalone has been investigated in both cardiovascular disease and cognitive impairment (39). In an add-on study to a large cardiovascular clinical trial, the Montreal Cognitive Assessment (MoCA) was performed at the time of randomization to apabetalone or placebo. There was an improvement in the MoCA score in those with baseline scores of 21 or less, a prespecified analysis (40). This preliminary study suggests that treatment of cognitively impaired individuals with apabetalone or other BET inhibitors may benefit cognition and warrants further investigation.

### Telomerase

Telomers are composed of repetitive nucleotide sequences that form a cap which functions to maintain the integrity of chromosomes. Telomere attrition induces permanent cells cycle arrest and cell senescence. Study of human telomerase reverse transcriptase established that telomere dysfunction induces premature aging, cancer, and neurodegeneration. Telomeres function to maintain genome stability, tissue stem cell reserves, organ system homeostasis, and normal lifespan (41).

GV 1001, a reverse transcriptase inhibitor, has been studied in patients with moderate to severe AD. Participants exhibited an improvement on the Severe Impairment Battery not reflected in other measures (42). Further investigation of GV 1001 and other reverse transcriptase inhibitors may reveal important benefits of telomere protection.

A clinical trial of Emtriva^®^, an anti-retroviral reverse transcriptase inhibitor approved for use in treatment of human immunodeficiency virus (HIV) infection, is being studied for its possible utility in the treatment of AD. At baseline, patients will have mild to moderate AD; they will be treated for up to eight months. The primary outcome of this study is safety and tolerability. Secondary outcomes include inflammatory biomarkers, Mini-Mental State Examination, CDR, ADAS-cog, Alzheimer’s Disease Cooperative Study Activities of Daily Living (ADCS ADL) scale, Free and Cued Selective Reminding Test, and CSF amyloid and phospho-tau measures. Twenty-five participants will be assigned to active therapy and 10 to placebo in a Phase 1 study. This study will provide insight into the potential therapeutic benefit of reverse transcriptase inhibition in AD.

### Hormonal Therapies

Hormonal changes occur as part of the aging process and create a systems and CNS milieu that differs from that of younger individuals (43). Menopause is a marked change that occurs in women and may contribute to the increased risk of AD observed in older women (44).

Clinicaltrial.gov lists 10 interventional studies for estrogen or estrogen receptor agents. Nine of these have been completed. An active Phase 2 study will compare S-equal to placebo over a 24 month exposure with outcomes including drug-placebo differences on measures of arterial stiffness, white matter lesions on MRI, and change in cognitive score measured on the Preclinical Alzheimer’s Cognitive Composite. The relative lack of activity regarding estrogen replacement therapies for the treatment of AD may reflect the negative outcomes on clinical trials to date and difficulties designing trials particularly with regard to the timing of estrogen therapy relative to menopause (45).

Estrogen levels decline and follicle stimulating hormone (FSH) levels increase at the time of the menopausal transition. Recent preclinical observations demonstrate that FSH increases amyloid beta production, increases tau hyperphosphorylation, and impairs cognition in a transgenic model of AD. Blocking FSH ameliorated AD pathologic and behavioral phenotypes by inhibiting the gamma secretase pathway of amyloid processing (46). These observations suggest that further interrogation of treatment of elevated FSH in postmenopausal women, or prevention of FSH elevation in peri-menopausal women, may lead to new therapeutic interventions.

Testosterone levels are often lower in older men than in younger individuals and declining testosterone may contribute to cognitive decline and vulnerability to AD. Clinicaltrials.gov lists three trials of testosterone for the treatment of AD or MCI. Two of these studies have been completed. The third study is a Phase 3 9-month precision medicine approach assessing the impact of dietary supplements including hormones. Testosterone may be among the hormones included. Outcomes include drug-placebo differences in change on the MoCA, CNS Vital Signs Neurocognitive Index scores, Alzheimer’s Questionnaires 20 and 21, the Patient Reported Outcome Measurement Information System scores, discontinuation rates, and safety.

A 24 week double-blind placebo-controlled trial of testosterone therapy in men (mild AD dementia or normal elderly men) found no cognitive benefit with therapy but significant improvement on the caregiver version of the patient’s quality of life (47).

### Plasma Exchange and Parabiosis

Parabiosis (establishing a shared circulation between younger and older mice) or repeated injection of plasma from the young mice into older ones led to a marked restoration of synaptic and neuronal proteins, as well as an improved working and associative memory. No effect on amyloid plaques was observed (48). This approach to treating the biology of aging by exposure to young plasma is a rejuvenation strategy that depends on the multiple elements with possible pharmacologic activity in plasma.

Clinicaltrial.gov list three trials of plasma exchange for the treatment of AD, two of which have been completed, and three trials of treatment with plasma transfusion or use of plasma elements such as albumin. The feasibility of performing clinical trials with plasma infusions from younger individuals to participants with AD has been established in a double-blind crossover study with active therapy or placebo for four weeks, followed by a six week pause, and then a four-week exposure to the alternate therapy. Stability and safety of the intervention was demonstrated (49).

The Alzheimer Management by Albumin Replacement (AMBAR) trial involved 347 patients that were randomized to one of four treatment arms. Patients received six weeks of weekly plasma exchange with 5% albumin replacement. This was followed by a monthly low volume plasma exchange and randomization to one of four groups: 20 grams albumin, 20 grams albumin alternating with 10 grams IVIG, 40 grams albumin alternating with 20 grams IVIG, or placebo/sham infusions. Those in the plasma exchange group had significantly less decline on the ADCS ADL scale and a trend towards less decline on the ADAS-cog (the dual primary outcomes). Treated patients scored significantly better on the CDR-sb and the Alzheimer’s Disease Cooperative Study Clinical Global Impression of Change. Participants with moderate AD (MMSE score 18–21) had significant drug-placebo differences on the ADCS ADL scale and the ADAS-cog. Those with mild AD (MMSE score 22–26) showed no response to therapy (50). The goal of plasma exchange is to reduce systemic and brain chemokines and cytokines that may exacerbate brain inflammation in AD. Plasma exchange may also affect amyloid dynamics, and replacement albumin may have antioxidant effects (51).

### Stem cells

Most adult body organs have regenerative stem cells responsible for tissue homeostasis and repair. Stem cells lose their regenerative potential during aging, compromising the maintenance of tissue integrity and health (52). Direct infusion of stem cells is one means of restoring stem cell function and their regenerative capacity. Mesenchymal stem cells are the most used cells in these trials. Their main mechanism of action may be the secretion of neurotrophic and angiotrophic factors including neurotropic growth factor (NGF), vascular endothelial growth factor (VEGF), brain derived neurotrophic factor (BDNF), and insulin growth factor (IGF). These growth factors promote regeneration and repair, allow the existing cells to function more normally, and may assume the role of some resident cells. Mesenchymal stem cells reduce local inflammation and may be able to transfer healthy mitochondria to dysfunctional cells (53). In patients with aging frailty, mesenchymal stem cells improved physical performance and inflammatory biomarkers (54). Studies using mesenchymal stem cells in AD patients demonstrated treatment to be safe and well-tolerated and significantly improved fluid-based and imaging biomarkers (55, 56).

Clinicaltrials.gov lists 23 stem cell clinical trials for AD or for multiple conditions including AD. Of these, 5 trials have been completed and 9 are withdrawn or have not been updated in more than two years and are of unknown status. Nine trials are active. All of them use mesenchymal stem cells derived from adipose tissue, umbilical tissue, or bone marrow. Cells are administered intravenously, intranasally, or into the ventricles via an Ommaya reservoir. All studies are in Phase 1 or Phase 2 and emphasize safety outcomes. Studies including only AD participants incorporate cognitive outcome measures. Results of these trials will provide information regarding the ability to use mesenchymal stem cells to rejuvenate stem cells in the cellular environment as a therapeutic intervention for AD.

### Multi-Modal and Lifestyle Interventions

In addition to pharmacologic and stem cell interventions relevant to the geroscience perspective on AD drug development, exercise and multimodal lifestyle interventions can be an important source of benefit for aging individuals. Cognitive reserve has been defined as the ability of the brain to optimize or maximize performance through differential recruitment of brain networks or use of alternative strategies. Cognitive reserve is often inferred from success in activities known to be cognitively demanding, such as educational level and occupational complexity. Higher cognitive reserve may express itself as resilience manifested as a lower level of dementia-related cognitive dysfunction relative to an expected severity based on age, genetic factors, or other characteristics. Contributors to resilience include lifestyle factors such as cognitive and social activity, physical activity, healthy diet, moderate alcohol consumption, and abstinence from smoking (57, 58).

The Finnish Geriatric Intervention Study to Prevent Cognitive Impairment and Disability (FINGER) was a controlled, parallel group comparison of a multimodal intervention to general health advice. The intervention included diet teaching, exercise, cognitive training, and vascular risk monitoring. The primary outcome was the Neuropsychological Test Battery which showed a statistically significant benefit in favor of intervention (59). Physical exercise is an example of an intervention with multi-system effects including musculoskeletal, cardiovascular, pulmonary, digestive, and cognitive functions. Physical activity can improve cognition including executive functioning and memory, independent functioning in patients with MCI or dementia, and psychological health in dementia (60). Nonclinical laboratory studies have shown beneficial effects of physical exercise on synaptic plasticity, neurogenesis, angiogenesis, and autophagy mediated through growth factors such as BDNF (61). Elevated BDNF levels have been confirmed in randomized clinical trials of exercise in patients with neurodegenerative disorders (62).

Clinicaltrial.gov currently lists 231 interventional studies for exercise in AD. Ninety-two of these are listed as currently active. Learnings from these studies applicable to cognitive aging, MCI, and AD will help guide optimal intervention strategies involving multimodal approaches including both lifestyle changes and pharmacotherapy.

## Drug Development Strategies for Alzheimer’s Disease that Facilitate Understanding Therapies from a Geroscience Perspective

**Figure 2.**
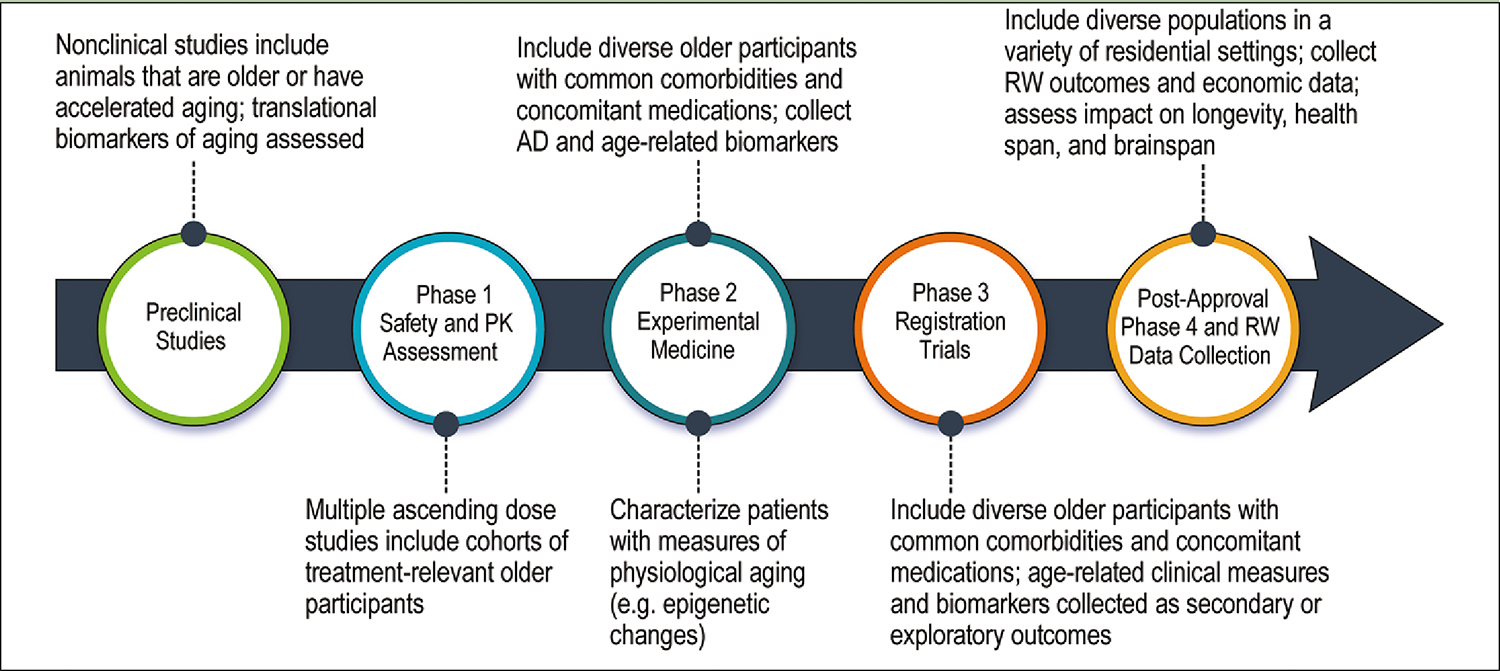
Alzheimer’s disease drug development with adjustments to increase the ability to learn about the influence of and impact on aging biology

The geroscience perspective can be comprehensively integrated into drug development programs that anticipate treatment use in older individuals. Adjustments can be made in non-clinical assessments, clinical trial design, biomarker choice, and outcome measures (Figure 2).

### Non-Clinical Assessment

Many non-clinical studies have been conducted in amyloid-related transgenic (tg) mice with the intervention timing based on when amyloid appears in the brain. Development of prevention strategies inaugurates treatment before amyloid accumulation begins; treatment strategies target amyloid after plaque occurrence. The age of therapy in mice is determined by the age at which amyloid accumulation begins in the tg species used and aging itself is not typically studied in these mice.

A variety of tools have been developed to assess aging, life span, and health span in older mice. Assessments can include many parameters that may be affected by aging including behavior and cognition, sensory function (hearing, sight, olfaction), body composition, kidney function, hematopoiesis, immune function, and gross and fine motor abilities (63). This battery of tests allows a comprehensive multiorgan system assessment of mice that could be used in the evaluation of therapeutic responses in mice prior to consideration of use in humans. The multidimensional assessment may provide important information regarding outcome measures relevant to aging that might be included in trials of AD therapeutics. A frailty index is available for mice, and its relationship to human frailty measures has been assessed and confirmed (64).

Accelerated aging in mice using models such as the senescence accelerated mouse (SAM) allows comparison of senescent-prone (SAMP) and senescent resistant (SAMR) strains. SAMP mice have accelerated aging and SAMR mice have normal aging. SAMP mice exhibit deficits in learning and memory, emotional function, circadian rhythm, and responses to pharmacologic interventions and well as pathological and biochemical changes (65). These mice present another platform for investigation of agents that may be used to treat AD where it is desirable to understand the effects of the agent on aging.

Aged rats can be used to assess memory and executive function and their response to treatment. Aged rats show impairments in strategy in the Morris water maze before they show impairments in learning (66). Such observations in animals allows dissection of therapeutic responses in animals and may provide guidance regarding assessment of older persons with AD. Etanercept, a tumor necrosis factor alpha (TNFα) inhibitor, was evaluated in aged rats exhibiting deficits in Morris water maze performance. Etanercept improved behavioral performance and changed protein expression (67), suggesting that this model can assist in assessing the impact of treatment on memory in older rodents and may identify protein expression biomarkers relevant to evaluating the biological effects of the intervention. A frailty index has been developed for aged rats and may provide translational links to similar tools used in older humans and possibly applicable as trial outcome measures (68).

Memory testing in non-human primates reveals that some aged monkeys are impaired while others perform as well as younger animals. Monkeys can be divided into aged impaired, aged unimpaired, and aged “other” (between impaired and unimpaired) based on their memory performances (69). Assessment of therapeutics in aged primates with memory impairment may provide insights into the therapeutic response of animals that bear many similarities to humans.

### Phase 1 Single Ascending Dose and Multiple Ascending Dose Trials

Many single ascending dose (SAD) and multiple ascending dose (MAD) Phase 1 trials are conducted on healthy young volunteers. The goal of these trials is to characterize the pharmacokinetics and establish the safety of the new therapy and to determine a maximum tolerated or maximum feasible dose. Age-related changes that affect pharmacokinetics support inclusion of at least one cohort of elderly in the Phase 1 MAD trials to assess the impact on pharmacokinetics of age associated alterations in liver metabolism, renal excretion, volume of distribution, and bioavailability (70). The age range of the older cohort should approximate the age range of the patients to be included in clinical trials and to be treated with the drug if it is advanced to market. In cancer patients where older cohorts have been more thoroughly investigated, older patients showed no differences in therapeutic response or toxicity compared to younger patients included in Phase 1 trials (71, 72).

### Phase 2 Proof of Concept Trials

Phase 2 proof of concept (POC) trials for drug development in AD usually include patients with MCI or mild to moderate AD dementia diagnostically confirmed with amyloid biomarkers. Trial participants tend to be younger than the typical population of patients with AD (16). Patients with earlier onset of their AD have a higher burden of neurofibrillary tangles as shown by tau PET and lower levels of other aggregated proteins such as TDP-43 and alpha synuclein (73–75). These biological differences may impact the generalizability to the older AD population of observations made in clinical trials.

Proof of concept/experimental medicine trials allow inclusion of exploratory outcomes that, if promising, may be advanced for use in Phase 3. From a geroscience perspective, outcomes relevant to aging may provide important information about whether these agents affect peripheral and systemic biology or brain senescence. Table 2 provides a list of instruments that may give insight into therapeutic impact on common disorders of aging beyond the learnings from cognitive, functional, and neuropsychiatric instruments typically used in trials of AD. Tools commonly used in studies of comprehensive geriatric assessment include usual gait speed, hand grip strength, timed up and go test, 6 minute walk test, 400 meter walk test, and the Short Physical Performance Test (76, 77), all of which are measures of intrinsic capacity, a concept developed by the World Health Organization (WHO) for standardization (78). The Clinical Trial Frailty Index and Barthel Index are also commonly used measures (79, 80). These assessments have been shown to correlate with measures beyond the specific physical domain including inflammation, oxidation, and mortality.

### Phase 3 Clinical Trials

Instruments of the comprehensive geriatric evaluation explored in Phase 2 trials can be incorporated as exploratory outcomes in Phase 3 trials. Introduction of exploratory outcomes must be done judiciously since sites may be unfamiliar with these tools, training and certification are necessary, and they increase site burden. They are warranted if they provide additional valuable geroscience insight into the intervention. Directional outcomes in Phase 2 may become more robust in Phase 3 where sample sizes are much larger. Geriatric outcomes may be of importance in discussions concerning drug cost reimbursement and national coverage, since these measures often relate directly to function and quality of life, outcomes of substantial value to payers.

## Biomarkers for Alzheimer’s Disease Clinical Trials that are Sensitive to Non-Alzheimer Disorders of Aging

Drug development programs cannot be advanced without biomarkers. The context of use of the biomarker may be for risk assessment, diagnosis, monitoring, pharmacodynamic measurement, prediction, prognosis, or safety (81). The amyloid, tau, neurodegeneration (ATN) framework is well established and provides information on diagnosis, prognosis, and pharmacodynamic effects for use in clinical trials of treatments for AD (2). From a geroscience perspective, these AD biomarkers may be complemented by biomarkers related to aging. Geroscience biomarkers would assess the presence of biological changes of aging at baseline and the impact of therapy. A consensus panel developed recommendations for a biomarker battery to be implemented in the TAME trial and these can be enhanced by additional biological measures (25).

Physiological age and biological age often differ. Epigenetic biological “clocks” offer one means of assessing biological age. DNA methylation is an epigenetic measure of aging that can be used to determine the biological age of trial participants (82). Refinements on DNA methylation measures such as DNAm-PhenoAge may better correlate with inflammation, mitochondrial dysfunction, and diseases of aging (83). “Inflammaging” refers to the chronic inflammation through the aging process (84). Measures of inflammatory clock can be an indication of biological aging (85, 86). Telomere shortening has been regarded as a measure of biological aging; it’s sensitivity and utility may be exceeded by other epigenetic biomarkers (87). Trial outcomes are usually interpreted according to chronological age, and investigation of trial results by biological age may be revealing.

The microbiome is increasingly recognized as a key contributor to health and disease. Assessment of dysbiosis in the microbiome is a valuable measure of age-related changes and may be particularly important for trials of oral medications that directly affect and are acted on by the microbiome (88). Table 3 presents biomarkers that might usefully be included in AD clinical trials.

## Conclusion

The usual inclusive age range for participation in AD clinical trials is 50 to 90. This includes young-old (65 to 74 years), middle-old (75 to 84 years), and old-old (85 and above) individuals. This 40-year span encompasses an enormous range of age-related changes. Modern health care has compressed morbidity and postponed mortality creating a larger population of older individuals at risk for and manifesting AD (89). Aging is associated with variable deficit accumulation from robust and healthy aging to increasing frailty. Aging is not accounted for in current AD clinical trials, in part because participants in clinical trials are younger and healthier than the general population of patients with AD. Most treatment users will be older than those participating in clinical trials and more attention is required to understand the impact of treatment on older patients. A geroscience perspective can assist in planning clinical trials and drug development programs that address the needs and anticipated impact of treatment on older patients.

## Figures and Tables

**Table 1.** Agents in the Alzheimer’s disease drug development pipeline that do not target amyloid or tau and focus on biological processes recognized as common in aging

Target Category	Phase 1	Phase 2	Phase 3
Cell death		Deferiprone	
Circadian Rhythm		Seltorexant	Piromelatine
		Trazodone	
Epigenetic Regulators		Lamivudine	
Growth Factors and Hormones		CORT108297	
		GnRH	
		Leuprorelin	
Inflammation	CpG1018	AL002	Masitinib
	Emtricitabine	Bacillus Calmette-Guerin	NE3107
	IBC-Ab002	Baricitinib	
	Salsalate	Canakinumab	
	TB006	Daratumumab	
	VT301	Dasatinib + Quercetin	
		Lenalidomide	
		L-Serine	
		Montelukast	
		Pegipanermin	
		Pepinemab	
		Proleukin	
		Sargramostim	
		Senicapoc	
		TB006	
		Tdap	
		Valacyclovir	
Metabolism and Bioenergetics	Nicotinamide Riboside	Chinese Traditional Medicine	Metformin
	Tricaprilin	Dapagliflozin	Semaglutide
		Insulin	Tricaprilin
		Insulin + Empagliflozin	
		T3D-959	
Neurogenesis	Allopregnanolone	Allopregnanolone	
		Sovateltide	
Oxidative Stress	CMS121	DHA	Hydralazine hydrochloride
		Edaravone	Icosapent ethyl
		Flos gossypii flavonoids	Omega-3
Synaptic Plasticity/Neuroprotection	Centella asiatica	AL 001	AGB101
		Bryostatin 1	Blarcamesine (Anavex 2-73)
		CY6463	BPDO-1603
		Dalzanemdor	Fosgonimeton (ATH-1017)
		Edonerpic	Simufilam (PTI-125)
		Elayta	Tertomotide
		EX039	
		ExPlas	
		Fosgonimeton (ATH-1017)	
		Levetiracetam	
		MW150	
		Neflamapimod	
		Simufilam	
		Tertomotide	
Vascular Factors		Telmisartan + perindopril	
		Yangxue Qingnao pills	

**Table 2.** Instruments that could be used in Alzheimers clinical trials to collect information relevant to the impact of therapy on age-related disorders

• Barthel Index of Independence
• Short Physical Performance Battery
• Clinical Trial-Frailty Index
• Usual gait speed
• Hand grip strength
• Timed up and go test
• 6 minute walk test
• 400 meter walk test

**Table 3.** Biomarkers of potential use to assess age-related changes in participants in AD clinical trials (from Justice, 2018 with additions)

• Biological clocks ○ DNA methylation
• Inflammation ○ IL-6, CRP, TNFRII, inflammatory clock
• Mitochondrial function ○ GDF 15
• Nutrient signaling ○ IGF-1, insulin
• Kidney function ○ Cystatin-C
• Cardiovascular health ○ NT-proBNP
• Metabolic aging and glucose control ○ HGB A1C
• Multi-omic assessment ○ Genomics, transcriptomics, proteomics, metabolomics
• Gut-brain axis ○ Dysbiosis of the microbiome
